# Computational systems biology in disease modeling and control, review and perspectives

**DOI:** 10.1038/s41540-022-00247-4

**Published:** 2022-10-03

**Authors:** Rongting Yue, Abhishek Dutta

**Affiliations:** Department of Electrical and Computer Engineering, University of Connecticut, 371 Fairfield Way, Storrs, CT 06269 USA

**Keywords:** Systems analysis, Dynamic networks, Regulatory networks, Control theory, Computational biology and bioinformatics

## Abstract

Omics-based approaches have become increasingly influential in identifying disease mechanisms and drug responses. Considering that diseases and drug responses are co-expressed and regulated in the relevant omics data interactions, the traditional way of grabbing omics data from single isolated layers cannot always obtain valuable inference. Also, drugs have adverse effects that may impair patients, and launching new medicines for diseases is costly. To resolve the above difficulties, systems biology is applied to predict potential molecular interactions by integrating omics data from genomic, proteomic, transcriptional, and metabolic layers. Combined with known drug reactions, the resulting models improve medicines’ therapeutical performance by re-purposing the existing drugs and combining drug molecules without off-target effects. Based on the identified computational models, drug administration control laws are designed to balance toxicity and efficacy. This review introduces biomedical applications and analyses of interactions among gene, protein and drug molecules for modeling disease mechanisms and drug responses. The therapeutical performance can be improved by combining the predictive and computational models with drug administration designed by control laws. The challenges are also discussed for its clinical uses in this work.

## Introduction

**Fig. 1:**
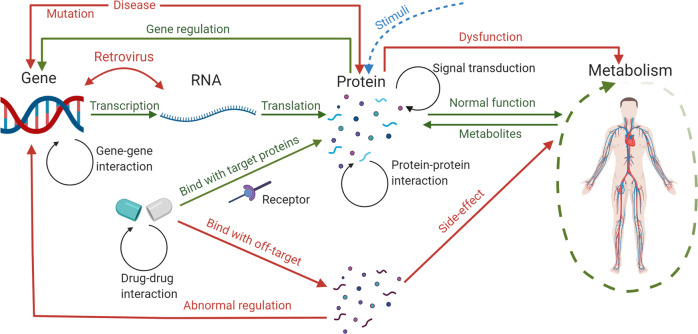
A systemic view of disease. Interactions among genomic, proteomic and transcriptomic levels reveal the regulatory process within organisms. Drug molecules intervene in this process by binding with specific target ligands. These omics data and chemical molecules are required to be analyzed simultaneously to study the entire disease mechanisms and drug reaction.

The high mortality of many diseases prohibits human longevity, and therapies need to be designed to suppress disease progression and aid organisms to recover from abnormal states^1^. However, the cost of launching new drugs is expensive and increasing, due to the long-term safety procedures in clinical trials^2^ caused by drug overdose toxicity and off-target side effects^3,4^. The unknown drug targets in individuals may also cause problems. For example, the drug Torcetrapib has been designed for cardiovascular disease^5^, but it may cause severe side effects of hypertension^6^. Analyses of omics, including genomics, proteomics, metabolomics and transcriptomics, contribute to the studies of disease mechanisms and drug responses. While a single omics layer focuses on a specific aspect with less complexity but limited information^7,8^. For instance, using only the conventional marker or the haplotype association cannot reveal the combined effects of Single Nucleotide Polymorphisms (SNPs), which potentially induces stroke^9^. The systemic view on dynamic gene regulation shows that genes work as part of complex networks instead of acting alone to perform cellular processes^10^. The integrative multi-layer omics data, such as transcriptional factors, genes and their expression products, provides a comprehensive map of metabolism and molecular regulation when analyzing and predicting based on complex cellular networks^11–15^. This leads to the prediction of potential molecular interactions through latent information of omics data. A general figure of omics data interactions is shown in Fig. 1.

In pharmacology, drug molecules act by binding to specific proteins, thereby changing their biochemical and biophysical activities^16^. Traditional treatment design based on physical parameters and external modalities^17,18^ or simple ligand-protein interactions^4^ are not sufficient for meeting clinical drug safety criteria or specifying variability among individuals. Modeling of the integrated clinical data and multi-layer molecular interactions makes the drug responses predictable^3,19^.

**Fig. 2:**
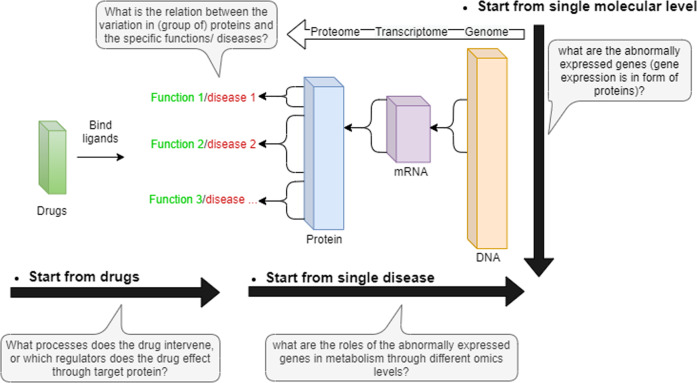
Analyses of disease and drug effect through single and multiple layers of omics data. Systemic view enables scientists to establish disease models from a higher hierarchic level by multi-layer data integration. Through vertical analysis within single layers, the disease-related molecules are identified by abnormal gene expression values. Through horizontal analysis with different layers, the interactive information are used to track diseases or drug effects throughout the entire biological process.

With multi-layer omics data, a single disease can be studied across different clinical modalities simultaneously (i.e., the horizontal direction in Fig. 2), and different diseases can be explored from a single modality (i.e., the vertical direction in Fig. 2). Systems approach makes chemical molecules and biomolecules more likely to be linked to phenotypes for analyzing diseases and drugs and identifying their potential connections. Modeling of disease pathways and drug responses through different layers of regulation contributes to drug repurposing and drug combination based on known molecular interactions. This review classifies the models for interactions among gene, protein and drug molecules into two main classes: static network and dynamic modeling. Both frameworks integrate biological information. The modeling is served for studying disease mechanisms and drug responses. The two main tasks include (1) deriving potential molecular interactions from disease mechanism and drug response, and (2) designing drug dosages.

## Network structure in systems biology

A network structure visualizes a wide range of components such as genes or proteins and their interconnections. Network-based modeling can be established for systematic analysis based on omics data from various scales^20^, which expands the use of bioinformatics beyond its original meaning by mining structural motifs for novel interaction prediction^21^. This agrees with the ideas that the networks with hierarchical bio-information consist of the metabolic, signal transduction, and gene regulation pathways all contribute to the analyses of interactions between protein inhibitors^22^. Diseases with overlapping network modules show significant co-expression patterns, symptom similarity and comorbidity^23^, whereas diseases residing in separated network neighborhoods are phenotypically distinct^24^.

A basic network is made up of nodes and edges. For molecular interactions, nodes can be genes^25^, proteins^26^, and drugs. Node annotations can be connective properties, including binding affinities^27^, interactive directions^28^, and the importance and confidence of the connections^29^. Edges link the nodes, and edge annotations can be functional interactions between nodes, including protein physical interactions, gene regulatory relations^30^, mechanism of activation and inhibition^31,32^, and disease associations^33^. Besides, network complexities reflect in its size. Large networks with high complexity can be iteratively divided into measurable subunits to reduce the complexity of analysis^34^, and each subnetwork can be a set of functionally grouped nodes^28^. The patterns in the known annotations can be used to predict new annotation^35^, and structural patterns can be obtained by network motif. Motif encodes regulatory behaviors and decreases internal cell noise^28^, and it also helps identify drug molecules with common reactions, discover unknown drug responses, and predict potential therapeutics^36^. The regulatory motifs in the gene regulatory network contribute to modeling the cell fate dynamics in the immune system^37^. The pathway motifs within gene regulatory networks help interpret genetic and epigenetic variation^38^. The topological motifs in the interaction network of drugs and targets help select target protein candidates for drug synergy^39^.

## Static network of diseases and drugs

A static network models the statically functional interactions from omics data. Network structure provides topological properties from the presented interactions. It integrates intra- and extra-cellular information for identifying the modules’ functional response by multiple network alignment. The overlapped multi-omics data integration is informative for reveal new molecular interactions^12,15^.

The purpose of constructing a static network is to predict the potential interactions among drug molecules and target proteins through the shared components, as they can be the intermediaries to convey information to different network layers^4,40^. For example, the diseases can be associated based on the shared genetic associations, the gene-disease interactions, and the disease mechanism^23,26,30^, such that disease connections can be built through the shared genes for drug repurposing. In a host-pathogen interaction network, the shared enzymes and regulatory components connect the metabolic reactions for predicting drugs for fungal infection^7^. Compared to a multiplex network, which only contains the same type of nodes and integrates the subnetworks from different layers, a heterogeneous network has the capability to include different types of nodes and edges. The multi-layer connections for the same nodes will result in a multiplex-heterogeneous network. More details about the different network structures can be found in reference^41^. Additionally, the new interacting pairs may account for variability in disease progression or drug response among individuals. To conclude, the shared components across layers may reveal new findings through multi-layer omics data modeled by a heterogeneous (or multiplex-heterogeneous) network structure.

The absent interactions don’t guarantee the negative interacting relations, since the available binding profiles are limited. One obstacle preventing expanding the database is that the clinical experiments are costly. To avoid the expensive clinic experiments, based on the static network models, machine learning-based methods are used to predict possible interactions using known interaction data.

### Interactions from omics data

Due to the expression relations, genes and proteins are always analyzed together for genetic analysis of complex diseases. For genome-wide association, proteins and gene interactions can identify densely connected modules in the human protein interactome^9^. The protein-protein interaction (PPI) networks encode the information of proteins (nodes) and their interactions (edges) into the network structure. PPI networks help predict the potential disease-related proteins, based on the assumption that shared components in disease-related PPI networks may cause similar disease phenotypes^7,33,42,43^. For example, PPIs can be used with gene co-expression networks to assess the host-pathogen response for clinical treatment of Covid infections^44^. To be specific, the HCoV-host interactome was used to predict SARS-CoV-2 pathogenesis and provide a theoretical host-pathogen interaction model for HCoV infections.

The aim of modeling static molecular interactions is to use the interaction profiles to find out the potential interacting pairs. The modeling starts from identifying disease-related regulators using omics data. Consider that the RNA-sequencing data on a disease-related microarray is available. The disease-related genes can be selected from the differentially expressed genes (DEGs) based on the moderated t-statistics analyses and empirical Bayes using Limma in R^45^. The genes with large variations in expression data can be chosen based on fold-change and p-value, and a PPI network can be mapped. Limma focuses on the statistical meaning of the gene expression level, and its performance is affected by the number of samples.

For gene co-expression analyses based on microarray data, Pearson Correlation Coefficient (PCC) is frequently used. For example, a gene co-expression network for the Z. mays and A. flavus genes can be mapped directly from pairwise PCC masked by a customized cutoff^46^. WGCNA^47^ constructs an approximately scale-free network for detecting functional gene clusters based on PCC of gene co-expressions, under the assumption that proteins work together to perform metabolic functions. The disease-related hub genes/proteins with high connectivity are selected from the clusters. However, *R*^2^ value and connectivity of the identified gene network are sensitive to gene quantity, and different parameter settings (i.e., soft threshold) will result in different co-expression modules. In the frequent gene co-expression network^48^, gene pairs with high PCC, which are collected from different cancer and normal microarray dataset, are selected to build subnetworks of tightly co-expressed gene clusters using an iterative greedy algorithm “Quasi-Clique Merger”. The edges in subnetworks are weighted by the frequency of these genes, and similar subnetworks are merged into larger networks that are identified for specific diseases. The researchers noted that compared to differential expression analysis, where normal samples are necessary for comparison, this approach integrates multiple microarray datasets that even make the use of data without normal samples, which makes the constructed network more informative. However, the size of the datasets has to be large enough to ensure a high level of significance for PCC. A decision tree-based method Randomforest GENIE3^49^ can be used to infer gene co-expression network by solving *p* (i.e., the number of genes) regression subproblems of identifying gene expression patterns and then grouping the genes. It can fast detect gene networks from large gene datasets that have multifactorial expression data. However, this method assumes knowing the transcription factors in the gene dataset of the experimentally confirmed gene interactions. Note that PCC assumes the gene expressions are linearly correlated, which may not be true for biology systems^48^. In a gene co-expression network for identifying cross-species interactions, mutual information and *Z*-scores of gene pairs are calculated using Context Likelihood of Relatedness algorithm^50^, which are used to infer edges in the network. As described by the authors, this algorithm can cope with nonlinear changes of gene expression, and it shows higher accuracy compared to PCC. However, PCC is still needed to discriminate the (positive or negative) directions of correlations of gene pairs. See Table 1 for comparison.

**Table 1:** Methods for constructing gene co-expression networks.

Algorithms and applications	Advantages	Potential limitations
Quasi-Clique Merger algorithm for finding co-expressed gene clusters^48^.	Integrates multiple microarray datasets, even including the data without normal samples^48^.	Requires large datasets to ensure a high level of significance for correlations of gene expressions.
Context Likelihood of Relatedness algorithm for inferring edges in the network to identify cross-species gene interactions^50^.	Captures nonlinear changes in gene expressions^50^.	Can’t discriminate the direction of correlations of gene pairs without Pearson Correlation Coefficient.
GENIE3 for inferring gene co-expression network^49^.	Fast detects gene networks from large multifactorial gene expression data.	Requires prior knowledge of the transcription factors.
WGCNA for detecting functional gene clusters^47^.	The approximately scale-free network structure reserves connectivity when randomly removing nodes.	Sensitive to the number of genes and the choices of parameters (i.e., soft threshold).

Eventually, the target proteins are obtained based on the gene clusters in the gene co-expression networks. Drugs that potentially intervene in disease progression can then be predicted based on these proteins. The question that remains is how to detect the disease mechanism relevant to small gene expression variations, since small changes in some genes may have more essential contributions to the overall process. Note that gene expression level study excludes the non-transcriptional interactions^48^, which can be a potential limitation for predicting molecular interactions.

### Drugs and targets interaction

Similarly, a static network can model the interactions among drugs and targets. Drug-target interaction (DTI) networks have been applied to study the prediction of drug response^51^, interaction profiles of new drug-target pairs^16,26,27,52^, and side effects of unknown drug combination^22,53,54^. A DTI-based target inhibition model has been proposed to identify the disease-specific target set with possible drug-target combinations by mapping drug inhibition profiles with the use of protein candidates, which were relevant to cancer survival with known drug binding profiles^52^. Notably, the drug target proteins in a DTI network may have larger degrees (i.e., more interactive molecules) than those proteins in a PPI network^16^.

**Fig. 3:**
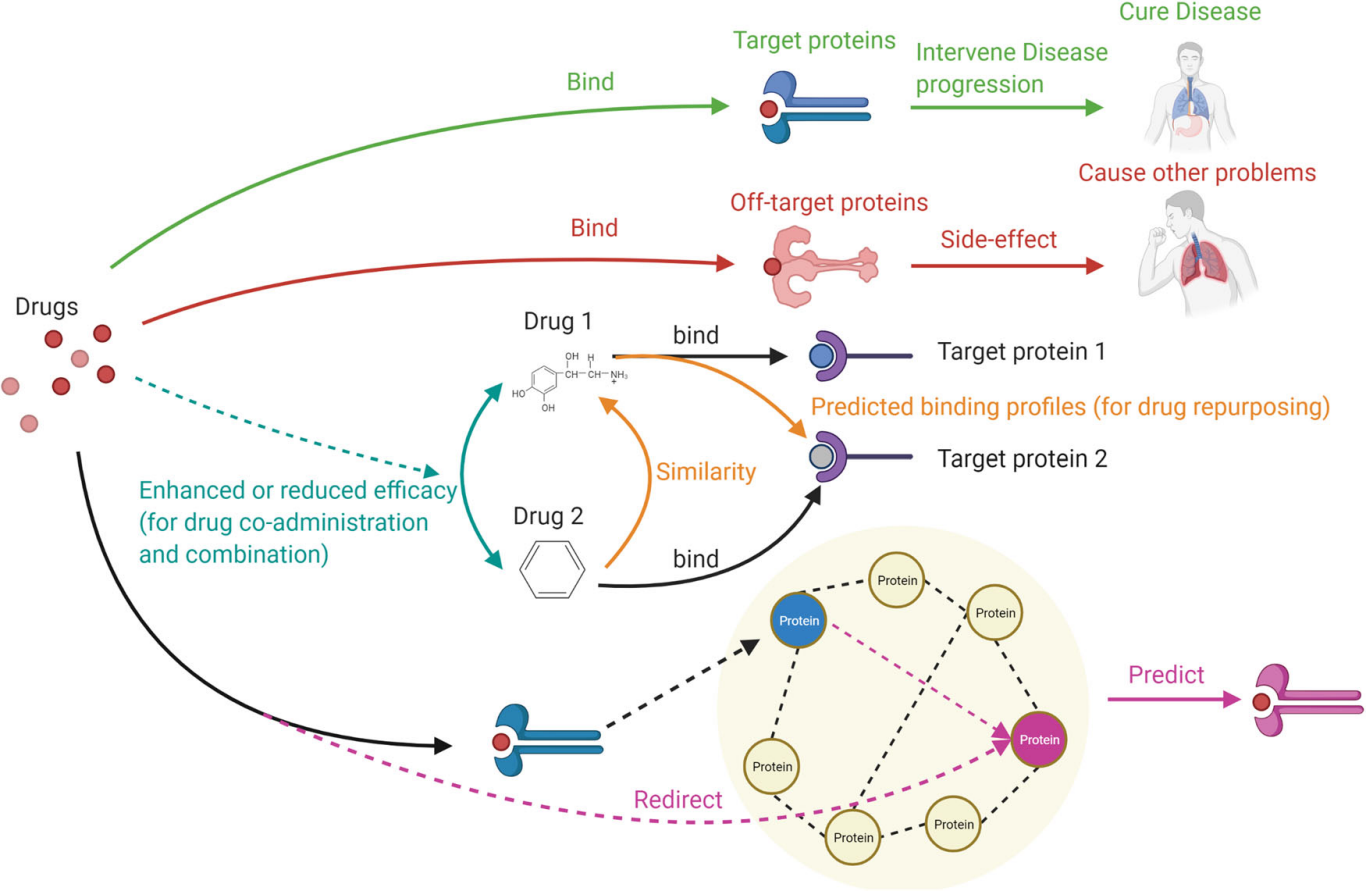
Interactions among drugs and target proteins offer chances for drug combination, co-administration, and repurposing. Drug molecules may bind with off-target proteins that induce side effects (labeled by red arrows), which should be avoided. Though, the therapeutic drug molecules should be reserved, as they function as desired to cure diseases (labeled in green arrows). Interactions between drugs provide an opportunity for enhanced therapeutic performance through drug co-administration and combination (labeled by cyan arrow). Drug similarity conveyed by drug-target interactions provides a chance for drug repurposing (labeled by orange arrows). Drug targets may be expanded to similar proteins using protein-protein interactions for drug repurposing (labeled in purple arrows). Drug molecules in this figure include epinephrine (for “Drug 1”) and benzene (for “Drug 2”) as examples, using the icons from Biorender.

Nodes in DTI networks include drugs, drug targets, and off-target proteins. For predicting drug side effects, the off-targets can be linked based on drug clinical relevance^54^. For drug combinations, different drugs may have interactions that induce side effects in patients or reduce the drug efficacy^55,56^. This requires us to identify potential drug-drug interactions (DDI), which can be explored by expanding DDIs through the shared targets of the drugs. The enriched DDIs identify potential targets and find new therapeutic uses (that the drugs do not initially aim at) and combination with surprising efficacy^4^. Figure 3 delineates a general view of the abridged drug and target interactions. Besides, the text mining-based predictions of molecular interactions rely heavily on the existing reports and references^57^, and insufficient clues would result in inaccurate predictions. Some of the frequently used databases for gene and protein interaction analyses are listed in Table 2.

**Table 2:** Databases for gene and protein interaction analyses.

Application	Database	Description	Reference
DTI, PPI	Comparative Toxicogenomics Database	Information of chemicals, pathways, disease, organisms, genes, drug-gene interactions. Data are mainly collected from references.	^39,53^
Gene regulation and interaction	GEO (Gene Expression Omnibus)	One of NCBI databases. Gene expression data (eg. RNA, genome methylation and proteins) that comes from data submissions such as microarray or other researches.	^13^
Genomics of cancer	Cancer Genome Atlas (TCGA)	Cancer molecular data including genome, epigenome, transcriptome and proteome.	^38^
Biological pathway	GeneGo MetaBase	Bioinformatics including signaling and metabolic pathways, interactions among drugs and proteins as well as kinetic information of drugs.	^54,65^
PPI, DTI, signaling pathway	KEGG (Kyoto Encyclopedia of Genes and Genomes)	Information of pathways, genome, chemicals and diseases based on diagrams of interaction and reaction. It is complementary to the majority of the existing molecular biology databases that contain information on individual molecules or individual genes.	^12,40,44,72^
PPI	STRING (Search Tool for the Retrieval of Interacting Genes)	Functional links in PPI based on experimental data. Interactions are predicted by comparative genomics and text mining based on the scoring system.	^9,44,85^
Gene regulation and interaction	CCLE (the Cancer Cell Line Encyclopedia)	Gene expression data for human cancer analysis, including information of mutation, Gene Methylation and the associations between cell line and genomics.	^51,52,170^
DTI, DDI	PubChem (NCBI)	Characteristics of chemical molecules and activities from experimental results or literature. For drug analysis, it provides information on the chemical structure for each drug and the validated chemical depiction information.	^51,85,95^
Gene regulation and interaction	GO (Gene Ontology)	Biological annotations including structure, function and dynamics in pathways, molecules and organism level for a variety of species.	^19,35,44^
DTI	STITCH (search tool for interactions of chemicals)	Profiles of chemicals and proteins interactions. The data source includes experimental results and text mining. More than 9 million proteins come from almost 2,000 organisms in this database.	^39^
DTI	ChEMBL	Biological activities and characteristics of molecules such as chemicals and proteins that contribute to the study of drug target and drug discovery.	^54,54,86^
Gene regualtion and transcription	UniGene (NCBI)	Gene sequences from animals and plants. The well-characterized sequences are driven from algorithm-based classification which helps to identify uniqueness among genes. The Source of intact gene sequences is GenBank.	^13,74^
DTI, DDI	Drug Bank	Drug-target and drug-drug interacting information such as chemical sequence, three-dimensional structure and pharmacological pathway involvement.	^16,40,71,76^
Genomics of breast cancer	METABRIC (Molecular Taxonomy of Breast Cancer International Consortium)	Clinical and expression data for breast tumors. The collected breast cancer specimens are grouped for discovery and validation. It helps to assess the survival prediction of cancer patients.	^173^
PPI	HPRD (Human Protein Reference Database)	Bioinformatics of human protein-protein interactions from literature and data are manually curated.	^33,42^
Signaling pathway	Reactome	Bioinformatics information including pathway, proteins and drugs for model visualization and analysis.	^9,29,44^
DDI	Online Mendelian Inheritance in Man (OMIM)	Disease data including disease loci, known disease genes and the known disorder-gene associations such as the molecular relationship between genetic variation and phenotypic expression.	^16,28^
PPI	Human Protein Reference Database (HPRD)	Protein information based on interactions described in published reports. The interaction set is expected to be biased toward known disease genes.	^15,33,42^

### An example of constructing a static network

**Fig. 4:**
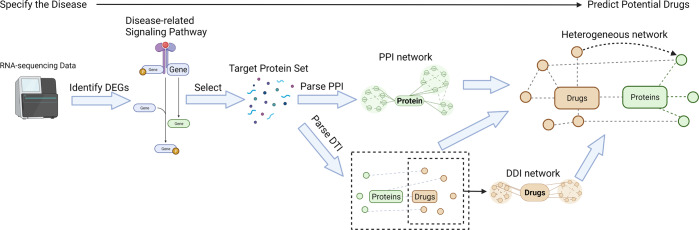
Statistic analyses on gene expression Values from RNA-sequencing data identify DEGs. The signaling pathways that contain the highest ratio of DEGs are regarded as disease-related. All or part of proteins in these pathways are selected to form the target protein set. By parsing PPI and DTI data from databases, the PPI network and the DTI network are constructed. The drug molecules in the DTI network are also used to parse and construct the DDI network. Finally, the DDI and the PPI are connected by the DTI, and thus the heterogeneous network is constructed. The task of predicting the potential drugs for a given disease is now transferred to the prediction of the interactions between the proteins and the drug molecules. Abbreviation: DEGs Differentially Expressed Genes, PPI Protein-Protein Interaction, DDI Drug-Drug Interaction, DTI Drug-Target Interaction.

Here is an example of constructing a heterogeneous network that integrates interactomics data from different subnetworks. The task is to predict potential drugs for a disease using RNA-sequencing data. Data can be obtained from online databases such as Gene Expression Omnibus database^58^. DEGs can be identified through statistical analyses (e.g., empirical Bayes using “Limma” in R^45^) on these gene expression data. The resulting genes are mapped into the signaling pathways database (e.g., KEGG database^59^) such that the highly perturbed disease-related pathways are selected. Target proteins are selected from the pathways, since we aim at intervening in the disease’s progress by blocking the relevant pathways using drugs. Interactions of target proteins are parsed from database (e.g., STRING database^60^) for constructing PPI networks and embedding the internal connecting information. DTI data is parsed from the drug database (e.g., DrugBank database^61^) by inputting the name of target proteins. Drugs that bind with at least one target protein are obtained to construct the DTI network. The DDI subnetwork is constructed by expanding the drugs from DTIs to their interactive partners, using interaction data from the drug database. And the internal connecting information among the expanded drug set is embedded into the subnetwork. The molecules from the three types of subnetworks are then integrated using a heterogeneous network, whose nodes are the drugs and target proteins, and edges are the interactions among proteins and targets. More recent works have focused on graph-based representation of molecular interaction network *G*(*V*, *E*), where *V* is the set of molecules and *E* is the set of molecular interactions^62–64^. And information is propagated from nodes to nodes through graph edges. The overview of the delineated process is visualized in Fig. 4.

### Limitation of static network modeling

The dynamic metabolic behaviors in patients result in changing expressions of genes. The interactomics in static modeling may become invalid, since molecule expression levels deviate a lot from the points that the model is built based on, thus leading to the failure of static modeling. This makes the dynamic modeling in Section V necessary. The major purpose of dynamic modeling is to map the regulatory relations among molecules, such that drugs can be used to intervene in disease progression by binding with target proteins, which drive the expression levels of genes to the normal range. Once gene expressions are corrected, the predictive static models will be valid again.

## Analysis of static modeling

Although the models of diseases or drugs have been studied for decades, the actual biosystems are far more complicated than complete modeling^65^, which indicates the potential of exploring more comprehensively models. This section reviews recent techniques that have been used to predict more information based on static models.

### Importance quantification

The importance of interactions in the network requires measuring and ranking for reducing network complexity and generating the weights, such that the simplified networks include only the most disease-related molecules^65^, which improves the efficacy of the learning process. The topological descriptors, such as degree, betweenness, and closeness, are frequently used to quantify the node importance in the network and embed spacial information into the modeling^16,66,67^. Node degree *D*, which is the number of connected edges^30^, measures the connectivity of nodes. In molecular interaction networks, the highly connected nodes (i.e., the hub nodes) usually provide more biological insights, compare to nodes with low degrees^45,47^. However, when identify disease-specific regulators, the importance will be penalized on hubs, since they don’t have much information^66^. The unconnected nodes will be discarded, since there is no path available to convey messages. Betweenness that describes the centrality of the given nodes is measured by the shortest path^68^, and it has more change when removing intermodular hubs compared to the intramodular^19^. Importance quantification can also be done based on statistics. For example, Z-score (i.e., the harmonic mean of precision and recall) measures the variability of the observations, and it has been used to quantify the importance of shortest paths between drug targets and the cardiovascular disease-related proteins^53^. The eigenvalues can be used to quantify the importance of data projection basis. In WGCNA^47^, the significance of gene co-expression clusters is quantified using the “eigen-genes” to reserve the most important genes and modules when modeling.

### Similarity analysis

Similarity characterizes how elements are similar to each other in a static network. The assumption is that similar nodes have similar interaction profiles. For example, similar chemical structures of drugs show similar therapeutic effects for diseases^33,40,51^. Different types of similarities can be used based on the type of molecules. Protein similarity can be obtained based on protein sequence using the Smith-Waterman alignment algorithm^27,69^. Similarities between small molecules drugs are often calculated based on Jaccard coefficient of chemical structure notated by Simplified Molecular Input Line Entry System (SMILES)^70^. Similarity between cell lines can be obtained using similarity of gene expression profiles^51^. Other types of similarities can be the drug phenotypic side-effect similarity^71^, pathological similarities^33^, and so on.

### Learning-based methods for clustering and classification

The evolution in learning-based networks has shown its ability to efficiently learn from massive datasets^4^. The learning-based methods can be supervised (with labels for training data), unsupervised (without labels) or semi-supervised (with partially labeled data).

**Fig. 5:**
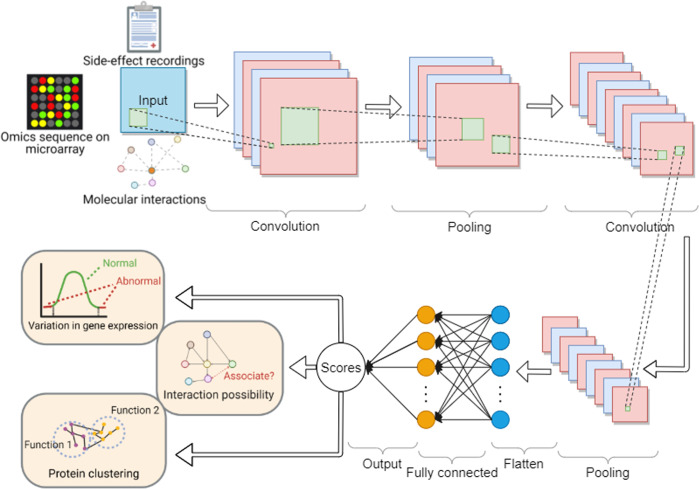
A convolutional neural network. This neural network is comprised of an input layer, convolutional layers (that extract features from hyperplanes of input data by projection/convolution), pooling layers (that reduce the spatial size and mitigate the locational sensitivity), flatten layer (that flattens the features and feeds them into the artificial neural network), fully connected layer (that learns nonlinear function of the extracted features) and an output layer. The input is the raw features such as molecules sequence patterns, gene regulation annotations and patterns, molecular interaction network motifs, molecular structures and structural associations, drug chemical structures, drug side-effect reports, and so on. The output can be the predicted classification (e.g., molecular binding profiles) and regression (e.g., quantified molecular binding affinities) when obtaining new samples.

A supervised learning approach enables the model to predict unknown parts (links) of the network based on the known interacting molecules^72–74^. Support Vector Machine (SVM), as a supervised learning model for classification, can refine topological information from network structure. SVM has been used for predicting DTIs based on DDIs, drug chemical structures, and side-effect information^75^. And the result shows that AUC values reach 0.76 in predicting the interaction between 261 drugs and 2,140 proteins. The multi-class SVM has been used to predict the therapeutic class of FDA-approved compounds using drug similarities, and it shows 78% classification accuracy of level 2 ATC codes among 410 drugs^76^. Kernels in SVM measure the features between gene pairs to train the classifier^77^, and the classifier makes the binary prediction for the interactions between the existing molecules and the incoming components^72^. Random Forest (RF), as an ensemble learning algorithm, can be used to performance classification based on decision trees^78^, such as the prediction of the contact probability between protein coevolved residues^79^. RF is robust to noise and it is capable to handle small sample size^80^. However, RF is less interpretable, and the computational load of RF will increase exponentially as the size of data increases^81^. The convolutional neural network (CNN), which uses the convolutional kernels and refines molecular features from arbitrary network frames of different sizes and shapes^82^, has been applied to refine DDI features using text mining on the biomedical information^56^. In the study, the prediction performance reached a F-score of 70% when evaluating about 900 drug documents. While a single-layer neural network may not have good predicting performance, a deep neural network can be deployed and obtain better results. Deep learning (DL) methods, which are built by multiple layers of neural networks, are gaining more and more attention because of the structural flexibility^83^ and their capability of extracting molecular patterns by mining latent information from the network structures^82,84–86^. For example, a deep CNN learning architecture^87^ shows its high concordance index (large value is better) in predicting drug-target binding affinity. While using more layers of neural networks results in more parametric settings, which could be potentially time-consuming. One efficient method to search these network parameters can be Particle swarm optimization^88^. A scheme of a convolutional neural network is shown in Fig. 5.

However, supervised learning heavily relies on the size of labeled samples during the training. Consider *n*_*d*_ drugs and *n*_*t*_ targets that include *n*_*d*_ ⋅ *n*_*t*_ drug-target pairs, then the number of available DTIs *n*_*a*_ < < *n*_*d*_ ⋅ *n*_*t*_. Moreover, when predicting DDIs base on drug side effect, the positive samples (i.e., drug pairs with known interactions) can be obtained from database, but the negative samples (i.e., drug pairs with with clinically validated safe co-prescriptions) are almost unavailable^89^. The lack of labeled data will decrease the predicting performance when using supervised learning methods. Though data preprocessing partly copes with the missing data problem, it causes certain information loss^90^.

Unsupervised learning aims at clustering data based on features, without the use of data labels. Examples are given as follows. When identifying relevance of disease phenotype and treatment response between patients, the number of patient clusters is unknown, and a hierarchical clustering (HC) algorithm with multiple linkage methods has been used for clustering patients based on genome-wise similarity and variability^91^. The benchmark test on 191 Multiple Sclerosis patient samples reaches Rand index moer than 0.85, and it also shows the capability of reducing feature dimension of Single Nucleotide Polymorphisms of 191 patients from more than 25,000 to about 1500. Advantages of HC includes that it is visualizable and user can customize the granularity by cutting the clustering at the desired level^92^. As a widely used unsupervised learning method, the autoencoder (AE) learns and refines the low-dimensional features from data to reconstruct the input, so that a minimalistic description can be derived for differentiability among samples^84^. This encoding-decoding frame can be used to denoise a single-cell RNA-sequencing model^93^, transform molecules directly into a numerical representation^86^, and compress computational dimensionality^4^ when detecting the cell type-specific clusters in an ensemble clustering way^94^. The stacked AE has been used to extract highly representative features from drug molecular structure and protein sequences, which help identify the potential DTIs^95^. Instead of capturing the deterministic latent features, the Variational Autoencoder (VAE) aims at capturing the distribution of the latent variables, with the help of variational inference^96,97^. Though VAE is capable of generating artificial samples for sparsely labeled molecules data, its heavy computational load due to the optimization of hyperparameters may limit the usage^92^. Note that AE and VAE are not classifiers.

Transductive learning learns to predict labels of unlabeled data in the existing network by training on entire dataset, while inductive learning learns a classifier that make prediction on testing data out of the current network. We focus on transductive learning, because the static network structure is deterministic for predicting potential interactions among existing nodes.

Semi-supervised learning (SSL) combines supervised and unsupervised learning. SSL is widely used for predicting molecular interactions using a small number of known interactions and many unknown interactions. Label propagation is a classical transductive SSL method that utilizes a small labeled dataset and predicts the label of the unlabeled data iteratively^98^. This method has been used to predict DDIs based on side effect similarity among 569 drugs with 52,416 DDI pairs^89^. The result showed that when the size of the training dataset is small, instead of the training data, the output of the proposed predictive model mainly relies on the geometric structure of the entire dataset. Also, the Area Under the Precision-Recall Curve value ranged from 0.650 to 0.729 for different ratios of testing and training dataset, which indicates the predictive model is stable. The autoencoder-based semi-supervised learning has been applied for predicting DTI^99^, DDI^100^ and PPI^101^. The unsupervised AEs/VAEs with a supervised deep neural network form a semi-supervised neural network, which shows higher AUC (over 0.8) compared to other learning methods^99–101^. Though, SSL cannot work without proper assumptions, as it will loss generalization from a finite training set to other test cases^102,103^. Besides, SSL is not always superior to supervised learning. An example is that a supervised logistic regression outperformed a semi-supervised label propagation when evaluating the prediction accuracy of gene functions, relevant disease and gene traits of on the full network connectivity, since the former algorithm efficiently extracts local network patterns, while the latter focuses on network topology^104^. The comparison of learning-based methods for predicting molecular interactions can be found in Table 3.

**Table 3:** Learning-based Methods for Predicting Molecular Interactions.

Type	Advantages	Disadvantages	Applications
Supervised learning	Use full label information of omics data.	Rely heavily on size of labeled data. Data preprocessing for noise and features may be needed, but this causes information loss.	Logistic regression for genome-wise prediction on relevant functions, disease and trait^104^; Multiple kernel learning for predicting drug response of cancer cell lines using omics profiles and pathways^90^; Support vector machine for drug-target interaction^75^; Convolutional neural network for identifying drug-drug interaction from document^56^; Random Forests for predicting protein contact^79^.
Unsupervised learning	No need for data labels. Suitable for the case where the labeled data is few and expensive to obrain.	Lose the informative features brought by labels.	Autoencoder for denoising a single-cell RNA-sequencing model^93^ and extracting representative features from drug molecular structure and protein sequences^95^; Hierarchical clustering algorithm for clustering patients based on genome-wise similarity and variability^91^.
Semi-supervised learning	Combine the benefits of feature extraction brought by unsupervised learning, and also make full use of the informative label data.	Algorithms work under proper assumptions. The trained model will loss generalization on testing data if assumptions don’t hold.	Autoencoder-based semi-supervised learning for predicting DTI^99^, DDI^100^ and PPI^101^; Label propagation for predicting DDIs^89^.

### Graph-based learning for predicting molecular interactions

Graph modeling is gaining increasing attention, since it encodes the structural and spatial information of data into models. The basic graph components include the sets of nodes, edges and an adjacency matrix that stores node connection information. A heterogeneous network of molecular interactions can be easily represented by a graph model. Graph Embedding (GE) encodes the spatial and topological information of graphs into low-dimensional feature vectors using a parametric function^105,106^. GE in transductive learning is deterministic because of a fixed graph. While for inductive learning, GE is generated from graph input features^105^.

Several graph learning methods have been proposed. Similar to CNN, graph convolution extracts features from graphs. Graph Convolutional Network (GCN), as a spatial convolution approach and the first-order approximation of Chebyshev polynomials of the graph spectral filter for semi-supervised classification tasks, aggregates the weighted node features from neighborhoods to the current node being visited using locally convolutional computation^107^. However, GCN has a relatively shallow structure. In a deep GCN, the oversmoothing property gives similar embedding to nodes from different labels/classes, resulting in mislabeling/misclassification issues^108^. The spectral graph convolution approaches partition/diffuse graph in Fourier domain using Eigen-decomposition of the graph Laplacian^109^, which could be computationally expensive when dealing with large graphs, and the graph convolution relies on specific graph structures that are not generalizable to other graphs with different structures^110^. Graph Attention network (GAT) utilizes the self-attention mechanism to obtain the normalized attention score (i.e., the relative importance) of each node from its neighborhood^110^, instead of the average aggregation functions in GCN. The attention mechanism solves the oversmoothing problem^108^. Node level attention is computed in parallel which is time-efficient. And it alleviates the effect brought by the lack of knowledge of the entire graph structure. However, GAT can’t tell the differences between local and global structures well because the aggregators lack of cardinality preservation mechanism^111^. Graph Autoencoder (GAE) and Variational GAE (VGAE) finds the (distribution of) latent variables of embeddings (which can be encoded by GCN), using a (variational) inference model and a Generative Model (GM), in low-dimensional space to recover the adjacency matrix of a graph in an unsupervised manner^112^. Loss functions in GAE and VGAE can be the reconstruction loss of the adjacency matrix (i.e., graph connectivity) and the variational lower bound, respectively^112^. The reduced dimensionality of data speeds up the training/backpropagation processes. However, the autoencoder-based methods focus on capturing most of the information from dataset based on a lossy reconstruction, which may not be relevant to the problem, and a relatively large dataset is needed to train an autoencoder. The inadequate bioinformatic data can be augmented by learning strategies. Graph Generative Adversarial Nets (GraphGAN)^113^ composed of a GM and a Discriminative Model (DM) can be used for this task. GM in both of GraphGAN and VGAE captures the distribution of the graph connectivity. However, rather than predicting the interacting pairs of nodes in each training epoch in VGAE, the GM in GraphGAN generates fake samples to deceive the discriminiator, such that DM learns to discriminate the true samples (that come from training data) from the generated samples. A Nash equilibrium that balances between two models is desired for convergence. Some limitations of GAN, including the unstable gradient updates and the vanished gradients of generator^114^, may potentially cause problems when being applied to graphs. See Table 4 for comparison of these graph learning methods.

**Table 4:** Graph learning methods.

Methods	Advantages	Limitations
Graph Covolutional Network^107^	Aggregate graph information and make the use of structural information of graphs.	Less computationally efficient for large graphs. Lack of generalization to graphs with different structure^110^. Oversmoothing of graph embeddings^108^.
Graph Attention Network^110^	Computationally efficient for node-level parallel processing. Don’t need the knowledge of the entire graph structure.	Can’t tell the differences between local and global structures well^111^.
(Variational) Graph Autoencoder^112^	Reduce data dimensionality and speed up the training process.	Captures more information from dataset, rather than the relevant information to the problem. And the reconstruction process loses information.
Graph Generative Adversarial Nets^113^	Can augment dataset and impute missing values.	Instability of gradient updates, and the vanished gradients of generator^114^, etc.

Additionally, the negative interacting pairs are rarely available in bioinformatics data, which results in an unbalanced dataset. The negative sampling^115^ can be applied that randomly assigns negative labels to the unlabeled data during the training process. This step aims at training the classifier to tell the difference between positive samples and the pseudo-negative samples drawn from training data. Lastly, a powerful package “PyTorch Geometric”^116^ (python), which contains multiple recent graph learning algorithms and uses fast tensor operation in GPU, can be used for implementation.

Compare to a binary classification problem (e.g., predict if the interaction exists or not), the multi-class classification problem is more attractive since more than two classes are involved. Examples of multi-class prediction can be found in predicting the interaction type of protein pairs^117^, the gene phenotype^118^, etc. The loss functions are designed based on class or labels. Each sample in a multi-class problem is assigned to one of the classes, and the labels for multi-label problems are usually obtained using categorical one-hot encoding^119^, which encodes the label as a vector with binary values. The entropy-based loss will be different for multi-class and multi-label classification. The Softmax function is often used to output probabilities of classes for the multi-class problems^120^, and Sigmoid function is used for multi-label problems.

The integrative static modeling conveys information through networks, which potentially results in a more comprehensive disease model by prediction. Similarities of nodes and edges among the nodes are calculated, and the quantified information is embedded in nodes and edges. Then the learnable models find representative features to predict potential molecular interactions. The benefits of static modeling-based prediction for disease treatment include: (1) the predicted disease-related regulators can be the new drug targets, which potentially improves treatment efficacy by finding new drugs for the same diseases; (2) the known drug target proteins redirect the drug molecules to bind with similar proteins through PPIs for drug re-purposing; (3) proteins/genes identified from the predictive model may explain variability among patients for specific disease phenotype or specific drug responses, which contributes to precision medicine; (4) the predicted off-target activities help avoid side effects; (5) the potential drug interactions contribute to designing novel therapies by drug combination, since the combinations may either reduce or enhance drug therapeutic efficacy. Though hypotheses and predictions can be made in a static network, the clinical use of potential drugs, drug combinations, or drug repurposing is highly concerned with patients’ safety, which means the accuracy and generalization of current predictive algorithms could be problematic. Besides, the commonly acknowledged problem in analyzing interactions between biological and chemical molecules based on database is data scarcity^20^, and how to use limited data to reliably produce more data for learning and obtain highly accurate predictive models remains an open problem.

## Dynamic modeling of regulation in organisms

The term dynamic disease refers to that disease pathogenesis is mainly caused by the appearance of new dynamic behaviors of organism, independently of the underlying pathogenesis^121^. The dynamics of diseases and drug reactions are informative for treatment design. For example, the viral reservoir dynamics are significant to understand the natural properties of ongoing viral progression with treatment^122^, which help researchers find the cures for diseases. A kinetic model predicts disease outcome by modeling the time-course behaviors of patients’ interactome^19,25^, and model performance can be assessed by its ability to generate testable predictions^123^.

### Gene regulatory network

**Fig. 6:**
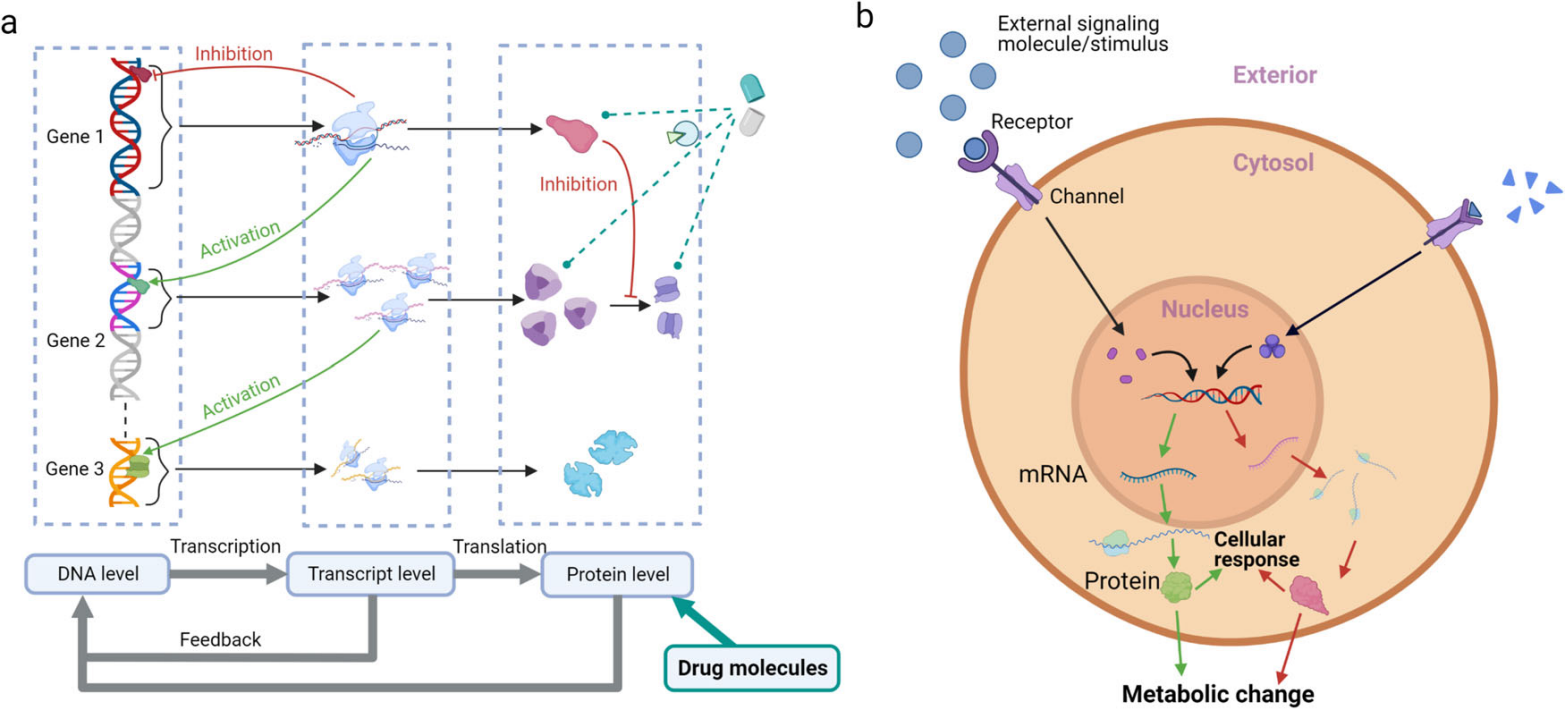
Schematic diagrams in dynamic modeling. **a** Gene regulatory network adapted from the work^25^. Gene 1, 2, and 3 are coding genes. Gene 1 regulates its own expression and those of Gene 2. The protein produced by Gene 1 regulates Gene 3 expression through a signaling factor/protein (that is produced from the protein expressed by Gene 2.) Drugs can intervene in the regulation by binding with proteins that change the gene expressions. **b** Diagram of signaling transduction. Signals are received and enter the nucleus to change gene expression. Proteins are synthesized to regulate phenotypic behaviors of cells or tissue. Errors (e.g., dysregulation) in signaling pathways (e.g., dysregulation) may cause the ceasing of cell apoptotic that results in unlimited growth and division.

In organisms, the changing gene expression results in phenotypic changes. Genes interact with each other through RNA and protein expression products, thereby governing the rates at which genes in the network are transcribed into messenger RNA^121^. The causal information in gene expression data, e.g., key drivers of complex traits in phenotype-related gene interaction network^13^, can be identified by variations in DNA and gene expression-related traits^25,37^. Gene regulatory networks (GRN) are thus used to detect potential mechanisms based on dynamic behaviors of epigenomic activity between normal and disease state^10^. For applications, GRNs have been used to analyze infectious diseases by detecting gene regulations related to the infectious and viral mechanism^15^. A combination of gene regulatory components including myeloid and lymphoid has been studied to identify cell fate specification^11^. Analyzing GRN is regarded as the reverse engineering for investigating gene regulatory relations by going backward with the observed gene expression^28^. The causal regulatory relations of genes are desired to be found from genome-wide expression data. A diagram of GRNs is shown in Fig. 6a. A GRN model contains genes and regulators as the nodes, and directional edges as the regulatory relations between the nodes^31^. To model and reconstruct GRN, the time-course microarray data of gene expression product (e.g., gene expression values, chromatin expression profiles) are required^25,28,124^, and the quantitative regulation can be obtained from computational models of GRN.

### Signaling pathway and transcription

Signal transduction pathway contains regulators between molecules in an organism. It attributes to changes in both gene expression and gene connectivity^29^. Errors in signal transduction lead to altered development and incorrect behavioral decisions in organisms, whose dysfunction may result in uncontrolled cell growth or tumorigenesis^28,125,126^. At the protein level, signaling pathways are comprised of protein interactions covering the biological functions in living cells, which captures the inter- and intracellular regulatory mechanisms of gene transcription and protein synthesis^28,31^. The modeling of dynamic signaling pathways measures disease progression. For example, pathway analyses contribute essentially to the systematic profiling of the transcriptome in heart failures^29^. The fungal signaling pathways model the regulatory behaviors in fungal pathogen infections^7^. Notably, the pathway-wide association can even extract valuable information from background noise and the context-specific logic of GRN^38^.

Transcription Factors (TFs) are the keys in modeling gene regulatory relation. For example, TF regulates the development of innate and adaptive cells of the immune system^11^. The dynamic transcriptional and translational subnetworks have been used to model the trigger mechanism of the innate response regulated by intercellular and intracellular heterogeneity^15^. A diagram of signal transduction is shown in Fig. 6b.

## Analysis of dynamic modeling

### Mathematical modeling for dynamic regulation

The dynamics of omics data reflects organism’s response to the changes of interior milieu or environmental factor. Variability in metabolisms and phenotypes can be huge even if most of the corresponding genes are the same^127^. Dynamic modeling exerts mathematical tools that quantify the rate of state change in gene regulation in different conditions and time sequences^25^. More logic models and kinetic models using Hill function or piecewise linear differential equations for quantifying the dynamic behaviors of gene network have been reviewed in the references^128^. This section reviews the computational modeling of diseases using Differential Equations (DEs), which can be used for drug administration by control theory.

DEs capture how the system reacts to the variations caused by disease or drugs. From a systemic view, DEs integrate information from multi-layer omics data into a unified form^34^. DEs model the dynamic behaviors of organisms, such as transcription^31^, gene regulation^25^, metabolite concentrations^129^, reaction rate^34^, and factors in signal transduction pathway^127^. DEs have also been used to quantify signal flow in pathways and explores the effect of oncogenic mutations on dynamics of ligands^123^. For disease modeling and treatment, DEs have been used to capture the dynamics of HIV viral infection^130^, drug response of an irradiation-induced cellular senescence^131^, and cancer cell population^132^. The components in DEs can be the metabolite concentration in nonlinear biochemical models^129^, the signal transduction molecules in dynamic cell compartment models^34^, and transcription factors and regulatory site^31^. A two-step approach, including the estimation of cell expression velocity using finite difference, and the estimation of a numerical square matrix that depicts the gene regulatory influence using sparse regression, can be used to computationally model a GRN from time-course single-cell RNA-sequencing data^133^. The accuracy of the approximated model depends on the user-defined sampling rate in finite difference. A robust dynamic model design makes it less sensitive to biological noise and disturbances, allowing us to track abnormal variations^21,134^. An example of the robust design of a physical system can be found in ^134^. Modeling noise is usually assumed to be Gaussian. Non-Gaussian stochastic noise can be modeled by solving the Fokker-Planck equation^135^.

### Parameter estimation in dynamic models

**Fig. 7:**
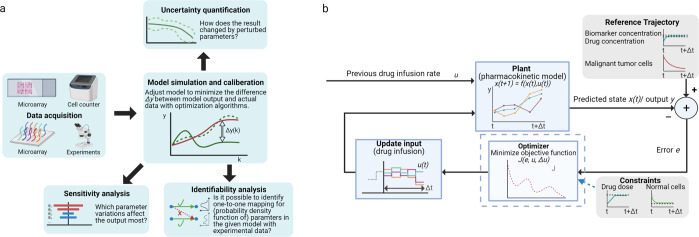
Dynamic modeling and analyses. **a** Scheme of parameter estimation. After data acquisition, parameters are fitted in models by minimizing the difference between experimental data and model output. Sensitivity analysis, uncertainty quantification and identifiability analysis help assess the performance and robustness of the fit. **b** Loop of Model Predictive Control. Based on the output of model prediction, this control strategy updates control input (e.g., drug administration) to make the system dynamics track reference trajectory (e.g., desired tumor cell decrement) during each time interval. Its essence is to handle the constrained optimization problem (Constraints can be maximal drug doses and minimal normal cell populations).

The main task of parameter estimation is to find the best-fit parameters to characterize physical processes and reproduce experiments^136–138^. To develop data-driven models, one can optimize the fit of a collection of parameters iteratively to a given dataset with random disturbance^137,139^, which explores the parameter space extensively and limits the number of non-convergent solutions^131^. The least-square estimation (LSE) that minimizes quadratic errors between the predicted model values and experimental data^129,140,141^), and the maximum likelihood estimation^142^, are widely used for this task. A Kalman Filter (KF) can recursively generate the maximum likelihood estimates for a linear dynamic system from a series of noisy measurements^143,144^. It handles the approximate modeling of high-dimensional noisy data with small sample sizes^145^. The Extended KF deals with nonlinear models in biology^146^. Note that the Gaussian noise is assumed in KF, and Particle Filters (PFs) are more appropriate when dealing with non-Gaussian processes. By randomly drawing samples from numerical simulation, the Monte Carlo method estimates parameter values^122^ and quantifies their uncertainties^147^. PFs in sequential Monte Carlo method obtain weighted samples from the non-Gaussian posterior probability of the state in nonlinear systems^148^. Study shows that a PF with an orthogonal basis (used for approximating the posterior by an orthogonal series expansion) outperformed Extended KF when estimating parameters of a Wiener anesthesia delivery model^146^. The process of parameter estimation is shown in Fig. 7a.

Sensitivity analyses assess how sensitive the models’ outputs are to the fitted models’ parameters changes. Sensitivity can then quantify model uncertainty through finite differencing or variational equations^139^. Identifiability checks the reliability of the estimates and assesses how well the model explains experimental data^144,149^. The global optima for parameter estimation are always desired, leading the multiple approaches to converge to the same solution ultimately^129^. Time of searching parameters is upper bounded to prevent an endless search^139^. Calling a single data cluster recursively may cause the absence of global parameters^34^ and get stuck in the misleading local optimum that should be avoided^139^ due to the generality. Precision medicine requires the patient-specific parameters for individual variability, resulting in a more complicated model. Note that a robust model design^134^ is desired after adding new parameters.

### Drug administration with control theory

Drug dosage is designed for patients’ recovery by control algorithms. Though the dynamics of drug concentration in plasma and drug response are usually nonlinear in real world, a linearized model is usually used to approximate the nonlinear behaviors of the original system due to the reduced complexity^150–153^. Several methods can be applied for the linear approximation. For example, when the pharmacokinetic data is available, drug model can be fitted using regression methods^4^. Linear models can be approximated from a nonlinear model by truncating Taylor expansion at the first-order term. Or, the linear descriptors can be obtained using Koopman operator, which projects the infinite-dimensional time-evolving observables (e.g., time-series data of drug concentration and cell population) into finite-dimensional states, using dynamic mode decomposition^154^. The Koopman method is driven by data, which can be generated from the nonlinear model^155^. Then the linear controllers for drug dosages can be designed based on a linear model.

The Proportional, Integral and Derivative (PID) control is a classic control algorithm that takes the error *e*(*t*) between closed-loop feedback signal *y*(*t*) and the setpoint *r*(*t*) (i.e., *e*(*t*) = *r*(*t*) − *y*(*t*)) as the controller input to calculate input needed, such that the model states can be driven to the setpoints^156^. PID controller has been used to design the drug administration for the chemotherapy treatment in a cancer model^18^ and the anesthesia in the neuromuscular blockade models^146,151^. The clinical evaluation and simulation results show that drug concentration levels can reach and be maintained at certain levels in acceptable time horizon. A PID controller copes with the uncertainty in the system’s dynamics caused by interpatient variability^157^ and time variations^151^. The concise scheme of PID^158^ makes it flexible for functional expansion, such as an I-PD controller constructed by cascading^18^. However, the performance and the robustness of PID controllers depend heavily on tuning^146,159^, and one of the frequently used tuning approaches is the Ziegler-Nichols method^160^.

Besides maintaining the drug concentration at certain levels, drug dosage should also balance the therapeutic performance and the toxicity/side effects. Optimal control law minimizes control errors (e.g., drive the systems along trajectories) and control efforts (e.g., less energy consumption)^161^ subject to system dynamics. The linear quadratic regulator (LQR), as one of the optimal control strategies, can be used for deriving an optimal control sequence (e.g., a sequence of drug infusion rates) when dealing with a linear model in the form $\dot{x}(t)=A(t)x(t)+B(t)u(t)$, with quadratic objective functions in form of *l*2-norm ∣∣ ⋅ ∣∣_2_) from time *t*_0_ to *t*_*f*_ with the initial state *x*_0_, as shown in Eqn. (1)^162^.

1 $$J=\frac{1}{2}| | x({t}_{f})| {| }_{{S}_{f}}^{2}+\frac{1}{2}\int\nolimits_{{t}_{0}}^{{t}_{f}}(| | x(t)| {| }_{Q(t)}^{2}+| | u(t)| {| }_{R(t)}^{2}),{{{\rm{subject}}}}\,{{{\rm{to}}}}\,\,x({t}_{0})={x}_{0}$$

where *S*_*f*_ is the terminal weighting matrix. *Q*(*t*) and *R*(*t*) are user-defined positive semi-definite and positive definite weighting matrix, respectively. Large *Q*(*t*) results in aggressive drug doses, and large *R*(*t*) leads to medical conservatism. The optimal feedback control law *u*(*t*) = − *K*(*t*)*x*(*t*) is derived by solving the algebraic Riccati equation and Hamiltonian. Compared to PID control, optimal control balances the drug toxicity and dosage. For chemotherapy treatment, the control problem can be to minimize the kinetic energies of all the cancerous cells with low drug toxicity^18,163–165^ by searching the optimal sequence of drug dosages. In a HIV model, the goal can be to maximize the benefit based on levels of healthy CD4+ T cells and immune response cells by reducing the systemic cost of anti-HIV drugs^130^, and the cost function consists of beneficiary T Cell population and systemic costs of therapy^166^. For multi-drug treatment, the ratio of co-administration between different drugs is also considered^164,167^. More examples of optimal control in cancer treatment can be found in the book^168^. The control law calculated by minimizing the cost can be piecewise constants or linear in the finite time horizon^169^.

Drug concentration in patients is delicate. The dangerous therapeutic window of drug concentration in plasma determines whether the level is tolerable and the drug is effective for patients^17,170^. Thus, state and input constraints are necessary to a drug delivery system. Usually, constraints include the upper boundary of toxicity level and the certain therapeutic window for drug concentration and disease progression^18,167^. The constrained optimization problems require more complicated control algorithms. In a multi-objective genetic algorithm, the Pareto optimal sets have been used to search for lower values of the objectives simultaneously^18^. Nonlinear optimization can be solved by Bock’s direct multiple shooting method with a numerical solution on a fixed control discretization grid^167^. The steepest descent method can be used to search numerical solutions iteratively to minimize or maximize the cost function^164^, and the numerical solutions can be derived with Miser3/Matlab^132^. Model Predictive Control (MPC) handles the constraints in optimization problems and the mismatch between nominal and actual processes^169^, compared to optimal control. MPC solves a finite horizon open-loop optimal control problem to obtain control actions with predicted states from models. The local asymptotic stability of the control law is guaranteed when time horizon is sufficiently long^169^. The loop of model predictive control is shown in Fig. 7b. Besides, controllers can also “learn” from complex situations through iterative learning-based strategies to obtain optimal parameterized control signals^171^. The equilibrium in a chemotherapy model refer to the elimination or the stop of cancer cell proliferation, so that no more treatment will be needed^164^. While using MPC, the feasibility and stability should be carefully considered. Compared to optimal control, MPC handles the constrained optimization problems, which makes it more suitable for the drug administration design subject to patients’ physical constraints.

The advanced control has good performance on drug dose adjustment. When eliminating cancer cell population, it has been found that giving bursts of high-dose abiraterone reduces tumor burden more than 10 times, compared to giving a constant dose^163^. For robustness, when model parameter error is 25%, MPC reaches 98% success rate (among 100 simulations) of stabilizing HIV infection in 2 years^165^. When designing the dosage of remdesivir for SARS-CoV-2, an optimal control sequence has been obtained by solving a constrained optimization problem, and simulation shows that the proposed control scheme reduces the treatment horizon from 10 days to 5 days and it also reduces more than 50% of the drug dose, compared to the recommended treatment regimes from FDA and WHO^172^. See Table 5 for comparing these methods used for drug dosage design. By using the advanced control algorithms, fewer drugs can be used to obtain the same or better treatment efficacy with less toxicity. There are some other control strategies that use feedback control laws based on nonlinear models. For example, a positive semi-definite Lyapunov function, whose gradient requires to be negative semi-definite, can be designed to calculate the controlled vaccination rate that asymptotically stabilizes Covid infection^138^.

**Table 5:** Control algorithms for drug administration.

Control Objective	Algorithm	Advantages	Disadvantages	Applications
Maintain drug concentration at certain levels	PID control	Easy to implement; flexible structure for functional expansion.	Design of PID controllers usually needs linear models. The linear approximation of the nonlinear model leads to information loss that eventually decreases control performance.	For anesthesia delivery^146^. For designing anti-retroviral therapy for HIV^165^.
Balance between drug toxicity, drug cost and therapeutic performance	Optimal control	Balance drug cost, toxicity and therapeutic performance.	More computations are required. Weighting variables *Q* and *R* are sensitive and they should be adjusted carefully.	For optimal imatinib treatment for leukemia model^174^. For eliminating cancer cells using abiraterone^163^.
Handle constraints in the model; Balance between drug toxicity, drug cost and therapeutic performance	Model predictive control	Consider physical constraints based on drug toxicity and patient conditions.	Solving the constrained optimization problem needs additional work to ensure stability, optimality and feasibility.	For stabilizing HIV infection^165^. For anesthesia delivery with uncertainty^175^.

Optimal drug dosage designed by control algorithms is used to efficiently intervene in disease progression, based on dynamic models with parameters estimated from sample data. Drug administration is transformed into an optimization problem with or without model constraints. The design of personalized treatments requires the patient-specific parameters for individual variability, which makes control scheme more complicated. Notably, the impulsivity of the model should be considered during the design process, and discrete drug therapy should be considered to allow the normal cells to rebuild in clinical treatment^166^.

## Conclusion and outlook

Launching new drugs could be costly compared to using existing drugs for new therapeutic performance, and drug predictions based on computational systems biology have shown its potential in precision medicine, drug combinations, and repurposing. A static network composed of molecular interactions is able to predict the potential interaction pairs based on known omics data by conveying information through nodes and edges. The new participants in the map of molecular interactions help obtain a more comprehensive view of disease progression and drug response. This results in using drug molecules with better therapeutic performance while avoiding off-target effects. Also, the potential patient-specific regulators can be identified to explain individual variability for personalized treatment. However, the predictive models may fail due to in vivo changing behaviors, which makes dynamic modeling necessary. Dynamic modeling aims at building math models to predict disease progression and drug response. Model parameters are estimated from clinic data. The potential participants identified from static modeling can be the new elements in dynamic models. By applying optimal drug dosages designed by control algorithms, disease progression can be intervened efficiently, which also indicates the predictive model in static modeling will be valid again. The combination of static and dynamic modeling makes it a powerful tool for disease analysis and therapy design. SSL outperforms other learning methods for making static modeling predictive, while the underlying assumptions may not hold in real cases, which makes the model loss generality. The learning algorithms that have an accurate prediction of other testing data are always desired. For drug administration, the simple control algorithms cannot meet the complicated design objectives (e.g., control with constraints), while a complicated control algorithm may not be time-efficient, though it aims at more control objectives. A control algorithm that handles multi-objectives and computes drug dosage needed efficiently is desired.

The modeling of DTIs by expanding drugs and targets based on DDIs and PPIs offer opportunities for (1) finding new targets for the same drug, (2) exploring new drugs for the same disease, and (3) minimizing off-target side effects for safe therapies. Though side effects should be avoided for safety issues, it does not mean all drugs that cause side effects should be abandoned. Treatments like chemotherapy harm normal cells, so we shall choose the drug agents that specifically target on cancer cells, rather than healthy cells. Combined with drug administration for changing conditions of patients using control theory, the treatment with better therapeutic performance and lower impairment can be realized simultaneously.
